# Supplementary material for: Top–Down Scoring of Spectral Fitness by Image Analysis for Protein Structure Validation
Source: J Chem Inf Model. 2025 Dec 19;66(1):567–76. doi: 10.1021/acs.jcim.5c02159 (PMC12801295; doi:10.1021/acs.jcim.5c02159)
Supplement: Supplementary file 1 [file ci5c02159_si_001.pdf]

Supporting Information

Top-Down Scoring of Spectral Fitness by Image Analysis for Protein Structure Validation

Benjamin D. Harding[1,3], Barry DeZonia[2], Rajat Garg[1], Ziling Hu[1], Katherine Henzler-Wildman[1,2], Frank Delaglio[5], Tim Grant[1,], Chad M. Rienstra[1,2,4,*]*

[1]Department of Biochemistry, University of Wisconsin-Madison, Madison, WI, 53706 USA

[2]National Magnetic Resonance Facility at Madison, University of Wisconsin-Madison, Madison, WI, 53706 USA

[3]Biophysics Graduate Program, University of Wisconsin-Madison, Madison, WI, 53706 USA

[4]Integrated Program in Biochemistry, University of Wisconsin-Madison, Madison, WI, 53706 USA

[5]Morgridge Institute for Discovery, University of Wisconsin-Madison, Madison, WI, 53706 USA

[6]Institute for Bioscience and Biotechnology Research, National Institute of Standards and Technology and the University of Maryland, Rockville, MD, 20850 USA

*Correspondence should be addressed to Chad M. Rienstra (crienstra@wisc.edu)

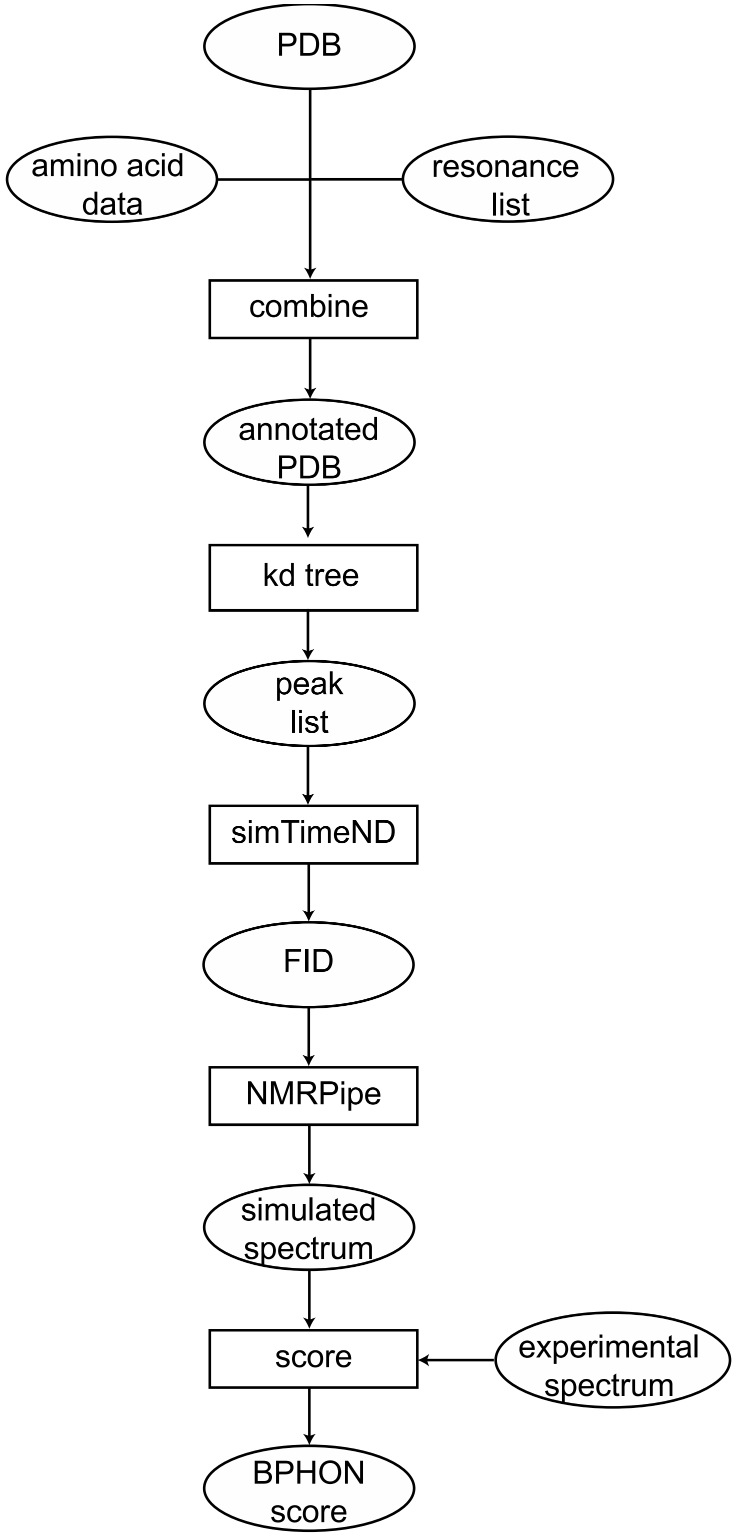

S1 Figure. BPHON pipeline flowchart. Ovals are inputs/outputs and rectangles are actions. The initial inputs by the user are a protein structure and a resonance list. Additionally, amino acid data, or hard-coded libraries of linewidths and scalar couplings are combined to create an annotated PDB. The PDB is then fed into a k-dimensional tree (kd tree) to generate a peak list which contains the atoms used to construct the peak, linewidths, scalar couplings, and peak height. The peak list is then the input to NMRPipe's program simTimeND, which appends header information from an experimental NMRPipe conversion script to the peak list to calculate a free induction decay (FID). The FID is then automatically processed using a default NMR processing script to generate an NMR spectrum in the frequency domain. The simulated and experimental spectra are then used as inputs to score their similarity using image analysis, yielding a final BPHON score.

experiment type	mixing time (ms)	cutoff distance (Å)
^{13}C-^{13}C	0-50	≤4
^{13}C-^{13}C	51-100	≤6
^{13}C-^{13}C	101-150	≤8
^{13}C-^{13}C	151-200	≤10
^{13}C-^{13}C	>200	≤12
^{15}N-$^{13}C\alpha$	-	1.6
^{15}N-^{13}CO	-	1.6

S1 Table. Default guidelines employed by BPHON for defining cutoff distances when constructing ^{13}C-^{13}C, ^{15}N-$^{13}C\alpha$, and ^{15}N-^{13}CO peak lists of peptides.

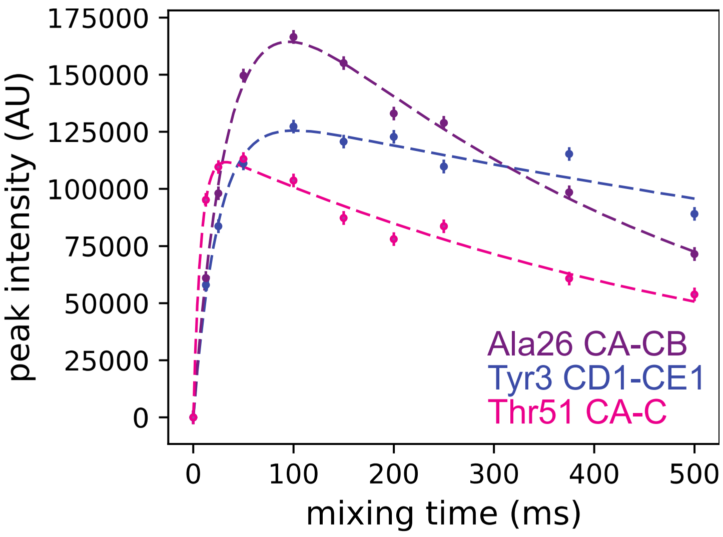

S2 Figure. Examples of three spins systems with different buildup rates used to fit to equation 1 in the main text. Ala26CA-CB, Tyr3 CD1-CE1, and Thr CA-C are spin systems that are representative of aliphatic, aromatic, and carbonyl spin systems.

Peak Position (w1)	Peak Position (w2)	I_{ij}	a	b
Aliphatic (0-80 ppm)	Aliphatic (0-80 ppm)	1	250	0.5
Aliphatic (0-80 ppm)	Aromatic (100-165 ppm)	1	50	0.5
Cα	Carbonyl (160-200 ppm)	1	3500	0.5
Aliphatic (0-80 ppm)	Carbonyl (160-200 ppm)	1	5000	0.5
Aromatic (100-165 ppm)	Aromatic (100-165 ppm)	0.5	250	0.5
Aromatic (100-165 ppm)	Carbonyl (160-200 ppm)	1	50	0.5
Carbonyl (160-200 ppm)	Carbonyl (160-200 ppm)	1	5000	0.5

S2 Table. Table of parameters from equation 1 in the main text used calculate peak intensity based on location of the chemical shift.

amino acid	^{13}C atom	linewidth (Hz)
**ALA**	CA	50
**ALA**	CB	20
**ALA**	C	30
**ARG**	CA	50
**ARG**	CB	50
**ARG**	CG	50
**ARG**	CD	50
**ARG**	CZ	30
**ARG**	C	30
**ASN**	CA	50
**ASN**	CB	50
**ASN**	CG	30
**ASN**	C	30
**ASP**	CA	50
**ASP**	CB	50
**ASP**	CG	30
**ASP**	C	30
**ASX**	CA	50
**ASX**	CB	50
**ASX**	CG	30
**ASX**	C	30
**CYS**	CA	50
**CYS**	CB	50
**CYS**	C	30
**GLN**	CA	50
**GLN**	CB	50
**GLN**	CG	50
**GLN**	CD	30
**GLN**	C	30
**GLU**	CA	50
**GLU**	CB	50
**GLU**	CG	50
**GLU**	CD	30
**GLU**	C	30
**GLY**	CA	50
**GLY**	C	30
**HIS**	CA	50
**HIS**	CB	50
**HIS**	CG	30
**HIS**	CD2	50
**HIS**	CE1	50
**HIS**	C	30
**ILE**	CA	50
**ILE**	CB	50
**ILE**	CG1	50
**ILE**	CG2	20
**ILE**	CD1	20
**ILE**	C	30
**LEU**	CA	50
**LEU**	CB	50
**LEU**	CG	50
**LEU**	CD1	20

**LEU**	CD2	20
**LEU**	C	30
**LYS**	CA	50
**LYS**	CB	50
**LYS**	CG	50
**LYS**	CD	50
**LYS**	CE	50
**LYS**	C	30
**MET**	CA	50
**MET**	CB	50
**MET**	CG	50
**MET**	CE	20
**MET**	C	30
**PHE**	CA	50
**PHE**	CB	50
**PHE**	CG	30
**PHE**	CD1	50
**PHE**	CD2	50
**PHE**	CE1	50
**PHE**	CE2	50
**PHE**	CZ	50
**PHE**	C	30
**PRO**	CA	50
**PRO**	CB	50
**PRO**	CG	50
**PRO**	CD	50
**PRO**	C	30
**SER**	CA	50
**SER**	CB	50
**SER**	C	30
**THR**	CA	50
**THR**	CB	50
**THR**	CG2	20
**THR**	C	30
**TRP**	CA	50
**TRP**	CB	50
**TRP**	CG	30
**TRP**	CD1	50
**TRP**	CD2	30
**TRP**	CE2	30
**TRP**	CE3	50
**TRP**	CN2	50
**TRP**	CH2	50
**TRP**	CZ2	50
**TRP**	CZ3	50
**TRP**	C	30
**TYR**	CA	50
**TYR**	CB	50
**TYR**	CG	30
**TYR**	CD1	50
**TYR**	CD2	50
**TYR**	CE1	50
**TYR**	CE2	50
**TYR**	CZ	30
**TYR**	C	30

**VAL**	CA	50
**VAL**	CB	50
**VAL**	CG1	20
**VAL**	CG2	20
**VAL**	C	30

S3 Table. Linewidths (Hz) dictionary employed by BPHON to assign linewidths to ^{13}C atoms in peptides. Methyl, methylene, methine, and carbons with no directly bonded ^{1}H have linewidths of 20, 50, 50, and 30 Hz, respectively.

amino acid	peptide bond	scalar coupling (Hz)
**ALA**	CA-C	55
**ALA**	CA-CB	35
**ARG**	CA-C	55
**ARG**	CA-CB	35
**ARG**	CB-CG	35
**ARG**	CG-CD	35
**ASN**	CA-C	55
**ASN**	CA-CB	35
**ASN**	CB-CG	35
**ASP**	CA-C	55
**ASP**	CA-CB	35
**ASP**	CB-CG	35
**CYS**	CA-C	55
**CYS**	CA-CB	35
**GLN**	CA-C	55
**GLN**	CA-CB	35
**GLN**	CB-CG	35
**GLN**	CG-CD	35
**GLU**	CA-C	55
**GLU**	CA-CB	35
**GLU**	CB-CG	35
**GLU**	CG-CD	35
**GLY**	CA-C	55
**HIS**	CA-C	55
**HIS**	CA-CB	35
**HIS**	CB-CG	35
**HIS**	CG-CD2	75
**ILE**	CA-C	55
**ILE**	CA-CB	35
**ILE**	CB-CG2	35
**ILE**	CB-CG1	35
**ILE**	CG1-CD1	35
**LEU**	CA-C	55
**LEU**	CA-CB	35
**LEU**	CB-CG	35
**LEU**	CG-CD1	35
**LEU**	CG-CD2	35
**LYS**	CA-C	55
**LYS**	CA-CB	35
**LYS**	CB-CG	35
**LYS**	CG-CD	35
**LYS**	CD-CE	35
**MET**	CA-C	55
**MET**	CA-CB	35
**MET**	CB-CG	35
**PHE**	CA-C	55
**PHE**	CA-CB	35
**PHE**	CB-CG	35
**PHE**	CG-CD1	75
**PHE**	CG-CD2	75

**PHE**	CD1-CE1	75
**PHE**	CD2-CE2	75
**PHE**	CE1-CZ	75
**PHE**	CE2-CZ	75
**PRO**	CA-C	55
**PRO**	CA-CB	35
**PRO**	CB-CG	35
**PRO**	CG-CD	35
**SER**	CA-C	55
**SER**	CA-CB	35
**THR**	CA-C	55
**THR**	CA-CB	35
**THR**	CB-CG2	35
**TRP**	CA-C	55
**TRP**	CA-CB	35
**TRP**	CB-CG	35
**TRP**	CG-CD1	75
**TRP**	CG-CD2	75
**TRP**	CD2-CE2	75
**TRP**	CD2-CE3	75
**TRP**	CE2-CZ2	75
**TRP**	CE3-CZ3	75
**TRP**	CZ2-CH2	75
**TRP**	CZ3-CH2	75
**TYR**	CA-C	55
**TYR**	CA-CB	35
**TYR**	CB-CG	35
**TYR**	CG-CD1	75
**TYR**	CG-CD2	75
**TYR**	CD1-CE1	75
**TYR**	CD2-CE2	75
**TYR**	CE1-CZ	75
**TYR**	CE2-CZ	75
**VAL**	CA-C	55
**VAL**	CA-CB	35
**VAL**	CB-CG1	35
**VAL**	CB-CG2	35

S4 Table. The dictionary of one-bond scalar couplings (J_{CC}) employed by BPHON when calculating ^{13}C-^{13}C NMR spectra of peptides. Hybridized sp3-sp3, sp2-sp2, Cα-C’ have scalar couplings of 35, 75, and 55 Hz, respectively.

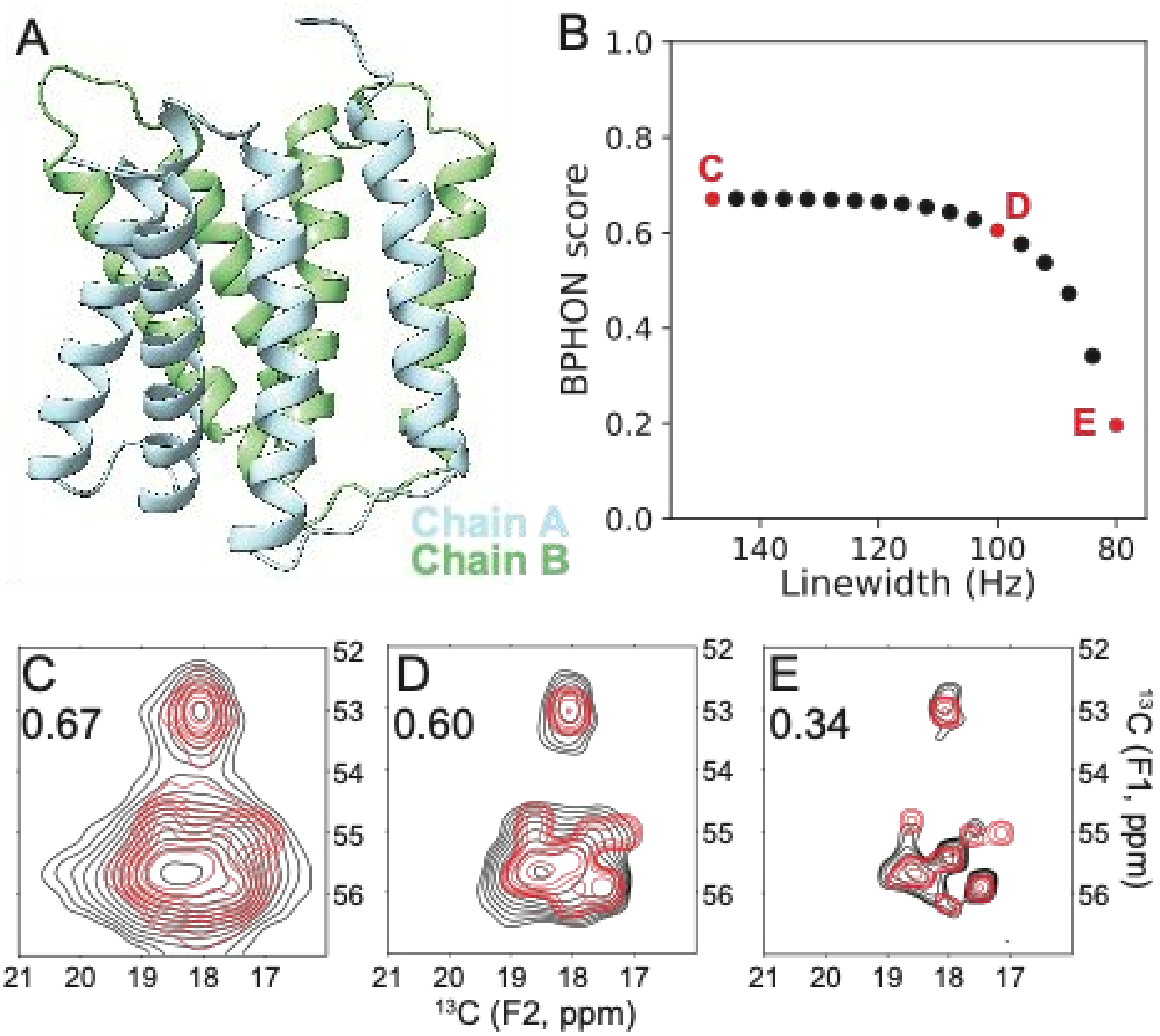

S3 Figure. BPHON analysis of the membrane protein EmrE. A) Modified structure of EmrE (PDB 8UWU) such that it is an asymmetric homodimer with the mutation S64V. This structure was chosen because the experimental conditions are similar to those used to generate the resonance list (BMRB 52793) and the mutations were made to match the amino acid sequence in the resonance list. B) BPHON scores of the entire (0-200 ppm) ^{13}C-^{13}C spectrum as a function of linewidth of an outlying Ala CA-CB crosspeak.
C-E) Overlay of simulated (red) and experimental (black) spectra of Ala CA-CB crosspeaks at 150 Hz (C), 100 Hz (D), and 80 Hz (E) linewidth. Similar to Fig 5 in the main text, the highest BPHON score is observed when the spectra are processed with larger linewidths. The experimental spectrum was collected at 750 MHz equipped with a 3.2 mm Balun probe spinning at 12.5 kHz. Spectra were simulated with the resonance list of uniformly labeled ^{13}C-^{15}N spectra collected below the phase transition (BMRB 52793).

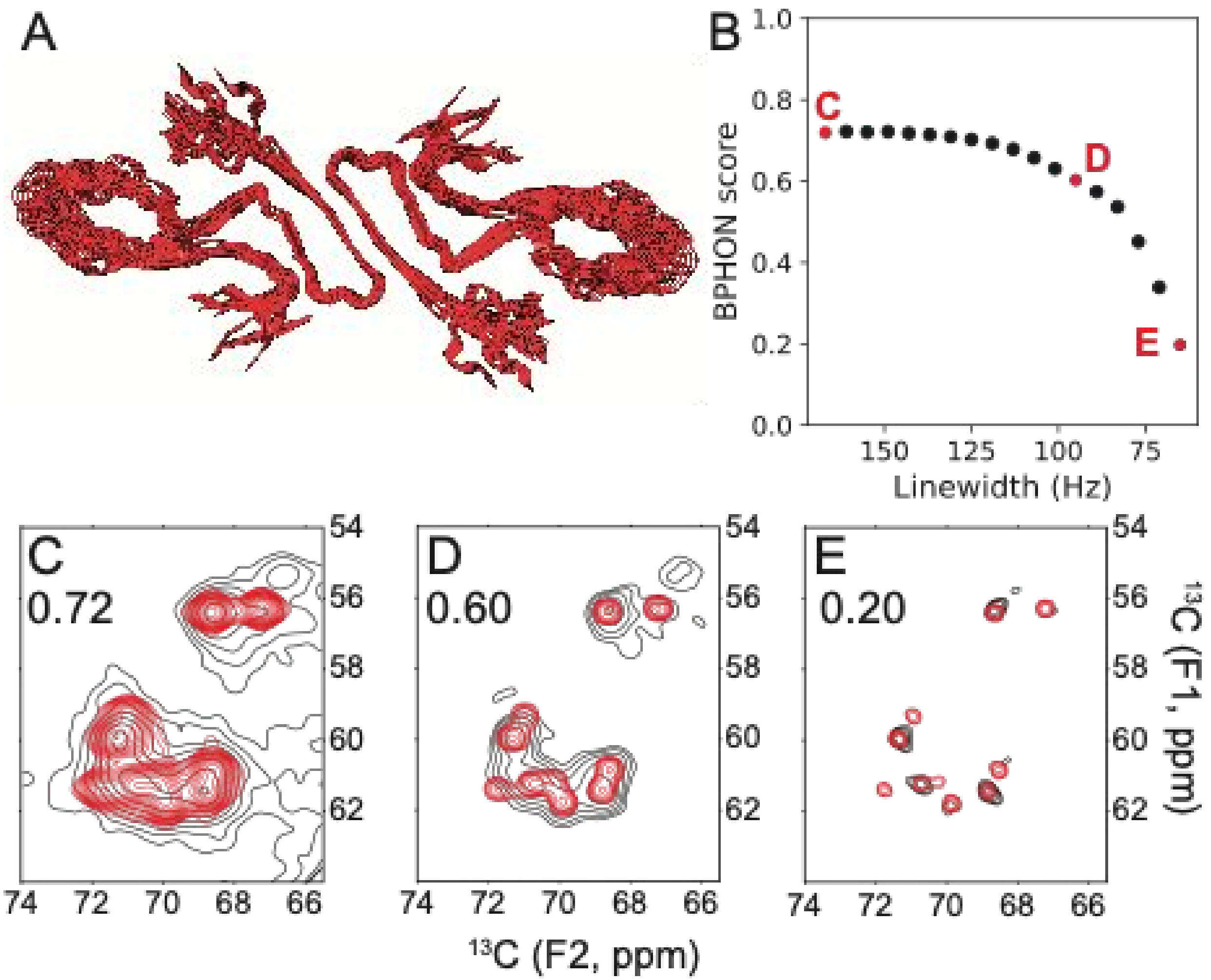

S4 Figure. BPHON analysis of alpha synuclein derived from a Lewy body dementia patient. A) Structure of Asyn (PDB 8FPT) derived from a patient with Lewy body dementia and calculated using SSNMR and its respective resonance list (BMRB 31068). B) BPHON scores of the entire (0-200 ppm) spectrum as a function of the linewidth of an outlying Thr CB-CG2 crosspeak. C-E) Overlay of simulated (red) and experimental (black) spectra of Thr CB-CG2 crosspeaks at 165 Hz (C), 95 Hz (D), and 65 Hz (E) linewidth. Similar to the trends observed in EmrE (Figure S3) and GB1 (Figure 5 in main text), the BPHON score is proportional to the linewidth of the spectra. The experimental spectrum was collected at 600 MHz equipped with a 1.6 mm Varian FastMAS probe spinning at 26.6 kHz.
