# Supplementary material for: Top–Down Scoring of Spectral Fitness by Image Analysis for Protein Structure Validation
Source: J Chem Inf Model. 2025 Dec 19;66(1):567–76. doi: 10.1021/acs.jcim.5c02159 (PMC12801295; doi:10.1021/acs.jcim.5c02159)
Supplement: Supplementary file 2 [file ci5c02159_si_002.pdf]

NMRFAM-BPHON

Operations Manual

NMRFAM
National Magnetic Resonance
Facility at Madison

UW-Madison

Version 1.0

TABLE OF CONTENTS

1. Introduction: 3
2. Installation: 3
3. Example Simulation: 5
4. Troubleshooting: 6

1. INTRODUCTION:

This manual is written for installation of BPHON (1.0) and related programs on a Ryzen workstation with 32 cores running Ubuntu 20.04.06.

2. INSTALLATION:

BPHON requires ChimeraX 1.7 or later), SHIFTX2 (1.13 or later), and NMRPipe (10.9 or later) to be downloaded on your local workstation. Download and install the following programs here:

- ChimeraX (1.7 or later): https://www.cgl.ucsf.edu/chimerax/download.html
- NMRPipe (10.9 or later): https://www.ibbr.umd.edu/nmrpipe/install.html
- Python 2
- SHIFTX2 (1.13 or later): http://www.shiftx2.ca/download.html
- NMRFAM-Sparky

Detailed procedures for downloading these programs are available in the Troubleshooting section.

The link to download BPHON can be found here: https://git.doit.wisc.edu/nmrfam-public/bphon-releases

This will give you a .whl file. In the ChimeraX command line, enter “devel install <path-to-whl-file>”

Successful installation of BPHON will yield the following in the ChimeraX log:

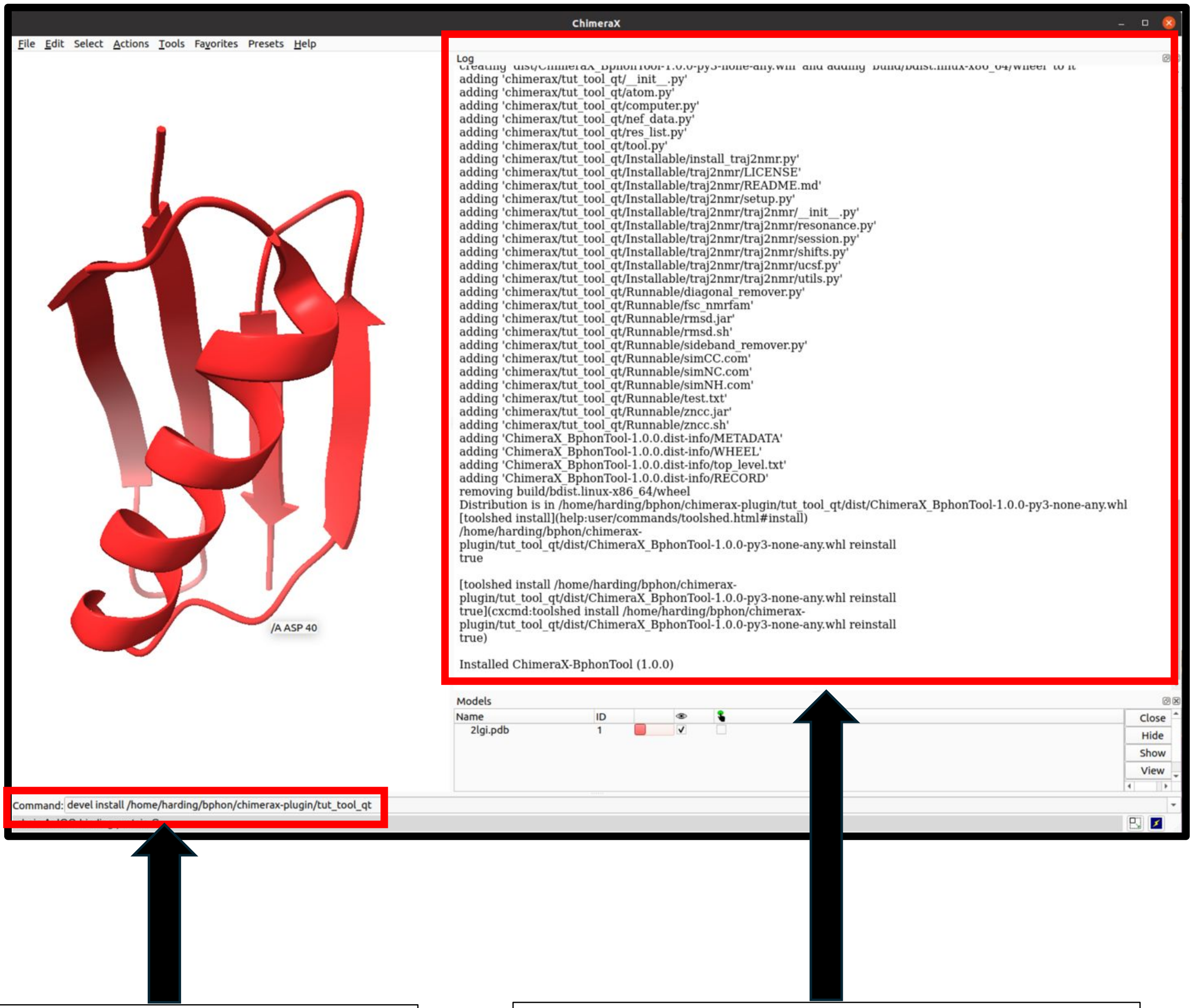

Example command to install bphon:

```
devel install
/home/harding/bphon/chim
```

ChimeraX Log upon installation will print everything BPHON installs and ends with "**Installed ChimeraX-BphonTool (1.0.0)**"

Figure 1. ChimeraX example installation command and ChimeraX log upon installation of BPHON (1.0.0)

3. Example Simulation

To simulate a spectrum, open a protein in ChimeraX. For this example, we use the protein GB1 (PDB 2LGI). **Be sure to select your protein**!

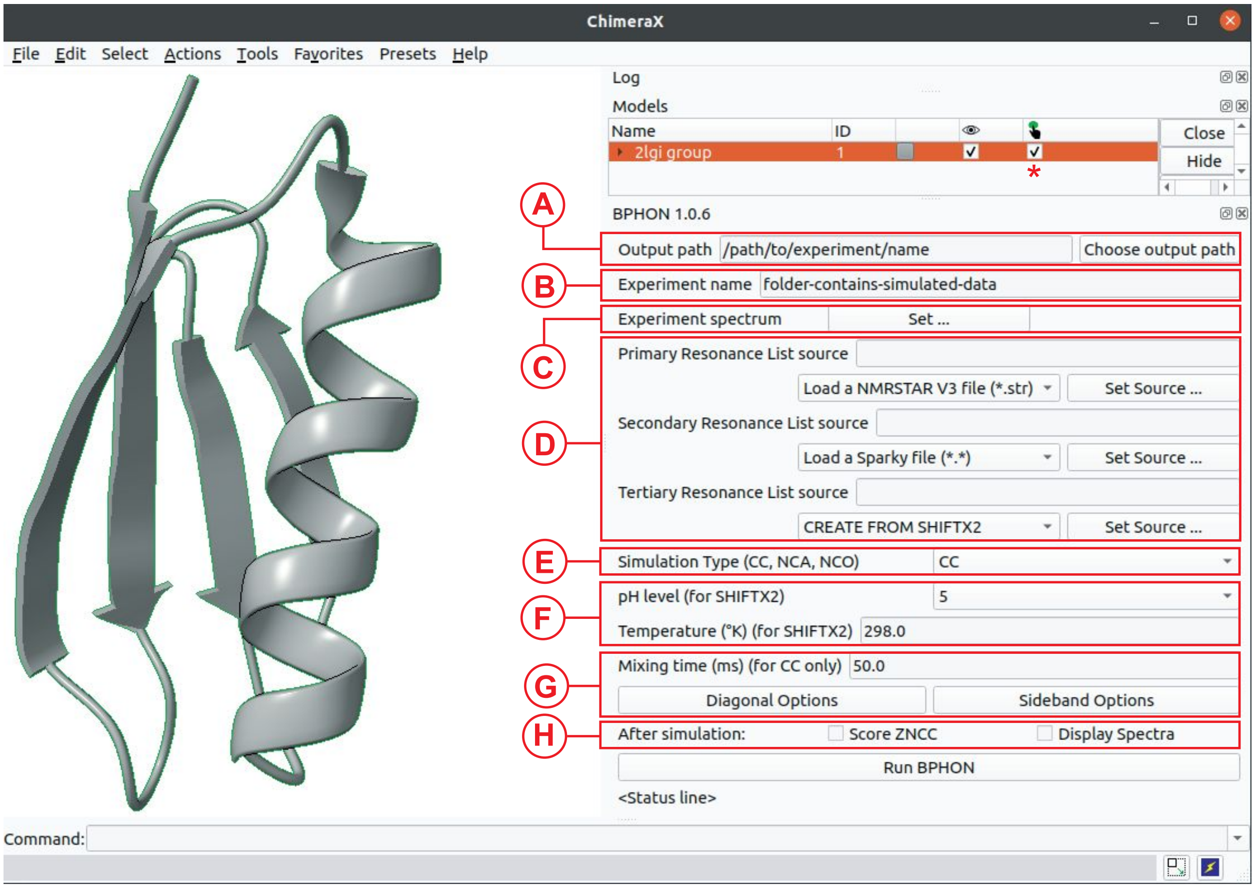

A: Choose a directory path where all the simulated data will be saved.

B: Name of the folder where all simulated data will be stored

C: Choose the experimental file the simulated spectrum will be scored against. We typically use a ^{13}C-^{13}C (DARR, 50 ms) spectrum collected at 14.1 T spinning at 26.6 kHz processed with a 59˚ sine bell offset (location: /mnt/nmrfam_data/taurus/2022/NOV22/120_CC_DARR_50ms.fid/CC50_experiment.ft2)

Examples of NMRPipe conversion and processing scripts are shown below for a ^{13}C-^{13}C (DARR, 50 ms) spectrum of microcrystalline GB1 collected at 600 MHz.

Conversion script:

```
#!/bin/csh -f

#Created by vrn2pipe

var2pipe -in ./fid -noaswap \
```

```
 -xN              3000  -yN            512\
 -xT              1500  -yT            256\
 -xMODE        Complex  -yMODE     Complex\
 -xSW        100000.00  -ySW      53333.00\
 -xOBS         150.556  -yOBS      150.556\
 -xCAR          97.438  -yCAR       97.438\
 -xLAB             C13  -yLAB          C13\
 -ndim               2  -aq2D       States\
 -temp            -5.0\
 -u1          -10000.0\
|nmrPipe  -out ./test.fid -verb -ov
```

Processing script:

```
#! /bin/csh -f


nmrPipe -in test.fid \
| nmrPipe  -fn LP -b -before -pred 2 -xn 256 -ord 8 -verb \
| nmrPipe  -out test.lp -ov


nmrPipe -in test.fid \
| nmrPipe  -fn SP -off 0.3 -end 0.995 -pow 1.0 -c 0.5   \
| nmrPipe  -fn ZF -size 8192                            \
| nmrPipe  -fn FT -auto   \
| nmrPipe  -fn PS -p0 -51 -p1 8.0 -di -verb \
| nmrPipe  -fn POLY -auto -window 32 \
```

```
| nmrPipe  -fn EXT -x1 200ppm -xn 0ppm -sw  -round 16 \
 -ov -out data.ft1
#
nmrPipe -in data.ft1 \
| nmrPipe  -fn TP   \
| nmrPipe  -fn SP -off 0.3 -end 0.995 -pow 1 -c 0.5 \
| nmrPipe  -fn ZF -size 4096                         \
| nmrPipe  -fn FT -auto    \
| nmrPipe  -fn PS -p0 -180 -p1 360 -di -verb \
| nmrPipe  -fn POLY -auto -window 32 \
| nmrPipe  -fn TP \
| nmrPipe  -fn EXT -y1 200ppm -yn 0ppm -sw -round 16 \
   -ov -out CC50_experiment-sine-0p3.ft2
```

Note: The phasing values (P0 and P1 in both dimensions) may be different. The bolded lines of the NMRPipe scripts are what BPHON will use to simulate and process its NMR data.

D: Resonance list. For this example, just used SHIFTX2 as the primary resonance list

E: Choose the default “CC” as the simulation type (Default)

F: Adjust pH and Temperature. Default values are 5 and 298 K, respectively.

G: Choose the mixing time (50.0 ms Default). Remove principal diagonal and spinning sidebands by typing in magnetic field strength (^{1}H Larmour) and spinning rate.

H: Choose “ZNCC” to score simulated and experimental spectra. Choose “Display Spectra” to view overlay of simulated and experimental spectra.

Click “Run BPHON”

The conversion file should be named “convert.com” or “fid.com” and the processing script should be named “CC.com”.

4. TROUBLESHOOTING

This section includes detailed procedures for downloading programs that interact with BPHON, including ChimeraX, NMRPipe, and SHIFTX2.

4.1 Downloading ChimeraX:

a) Go to the ChimeraX download page: https://www.cgl.ucsf.edu/chimerax/download.html. Find the **Linux version** and look for ChimeraX 1.7 (or later).
Alternatively, download it directly using `wget`:

```
wget https://www.cgl.ucsf.edu/chimerax/downloads/1.7/ucsf-
chimerax-1.7-linux_x86_64.tar.gz
```

b) Unzip the compressed file: `tar -xvzf ChimeraX-1.7-linux_x86_64.tar.gz`

4.2 Downloading NMRPipe:

a) we recommend first making a directory for NMRPipe (i.e. /home/Programs/NMRPipe)

b) in terminal: `cd /home/Programs/NMRPipe`

c) Download the required files from NMRPipe

```
wget https://www.ibbr.umd.edu/nmrpipe/install.com

wget https://www.ibbr.umd.edu/nmrpipe/binval.com

wget https://www.ibbr.umd.edu/nmrpipe/NMRPipeX.tZ

wget https://www.ibbr.umd.edu/nmrpipe/s.tZ

wget https://www.ibbr.umd.edu/nmrpipe/dyn.tZ

wget
https://spin.niddk.nih.gov/bax/software/talos_nmrPipe.tZ

wget
https://spin.niddk.nih.gov/bax/software/smile/plugin.smile.tZ
```

a) Download the following libraries in terminal:

```
sudo apt-get install tcsh
```

```
sudo apt-get install xterm
sudo apt-get install lib32z1
sudo apt-get install libx11-6:i386
sudo apt-get install libxext6:i386
sudo apt-get install xfonts-75dpi
sudo apt-get install msttcorefonts
```

b) Change shell to `tcsh`
c) In the NMRPipe directory, execute the `./install` script
d) Copy the following initialization scripts into ~/.cshrc

```
if (-e /usr/local/NMRPipe/com/nmrInit.linux212_64.com) then
        source /usr/local/NMRPipe/com/nmrInit.linux212_64.com
endif
if (-e /usr/local/NMRPipe/dynamo/com/dynInit.com) then
        source /usr/local/NMRPipe/dynamo/com/dynInit.com
endif
if (-e /usr/local/NMRPipe/com/font.com) then
        source /usr/local/NMRPipe/com/font.com
endif
```

e) In a new terminal window, execute "nmrPipe" and it should return the version of NMRPipe you installed. Example

** NMRPipe System Version 10.9 Rev 2021.258.11.26 64-bit **

4.3 Downloading Python 2:

a) Add the Universe repository

```
sudo add-apt-repository universe
sudo apt update
sudo apt install python2
```

b) Verify the installation

```
python2 --version
```

Note: Python 2 is no longer available in the universe repository for Ubuntu versions 24.04 and later. This is an installation guide for Ubuntu 20.04.

4.4 Downloading SHIFTX2:

c) In terminal, execute `sudo apt-get install python2.7 default-jre`
d) In a user-defined directory, execute `wget http://www.shiftx2.ca/download/shiftx2-v113-linux-20180808.tgz`
e) Execute `tar -xzvf shiftx2-v113-linux-20180808.tgz`

f) `cd shiftx2-linux`

g) In the code, change occurrences of “python” to “python2.7” by executing `awk '{ gsub(/python/, "python2.7"); print }' shiftx2.py > temp && mv temp shiftx2.py`

h) Allow the command to be executable by `chmod +x shiftx2.py`

i) Add the directory to your PATH variable by executing

   a. `export SHIFTX2_DIR=~/Programs/shiftx2-linux`

   b. `export PATH=$PATH:$SHIFTX2_DIR`

4.5 Downloading NMRFAM-SPARKY:

a) Instruction on how to download and install NMRFAM-SPARKY can be found at the following address: https://nmrfam.wisc.edu/nmrfam-sparky-distribution/

b) Upon installation, execution of the command “sparky” in Terminal should start the program.
